# Enabling Spectrally Resolved Single-Molecule Localization Microscopy at High Emitter Densities

**DOI:** 10.1021/acs.nanolett.2c03140

**Published:** 2022-10-21

**Authors:** Koen J. A. Martens, Martijn Gobes, Emmanouil Archontakis, Roger R. Brillas, Niels Zijlstra, Lorenzo Albertazzi, Johannes Hohlbein

**Affiliations:** † Laboratory of Biophysics, Wageningen University and Research, Stippeneng 4, 6708 WE Wageningen, The Netherlands; ‡ Department of Biomedical Engineering, Institute for Complex Molecular Systems (ICMS), Eindhoven University of Technology, 5600 MB Eindhoven, Netherlands; § Nanoscopy for Nanomedicine, Institute for Bioengineering of Catalonia, 08028 Barcelona, Spain; ⊥ Microspectroscopy Research Facility, Wageningen University and Research, Stippeneng 4, 6708 WE Wageningen, The Netherlands

**Keywords:** Single-molecule spectroscopy, multicolor imaging, single-molecule Förster resonance energy transfer (smFRET), stochastic optical reconstruction microscopy (STORM), point accumulation for imaging in nanoscale topography (PAINT)

## Abstract

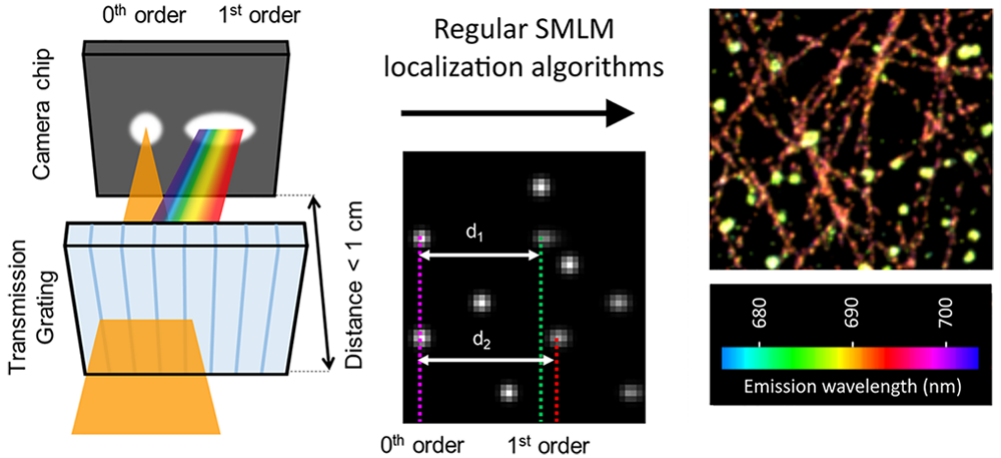

Single-molecule localization microscopy (SMLM) is a powerful super-resolution technique for elucidating structure and dynamics in the life- and material sciences. Simultaneously acquiring spectral information (spectrally resolved SMLM, sSMLM) has been hampered by several challenges: an increased complexity of the optical detection pathway, lower accessible emitter densities, and compromised spatio-spectral resolution. Here we present a single-component, low-cost implementation of sSMLM that addresses these challenges. Using a low-dispersion transmission grating positioned close to the image plane, the +1^st^diffraction order is minimally elongated and is analyzed using existing single-molecule localization algorithms. The distance between the 0^th^ and 1^st^ order provides accurate information on the spectral properties of individual emitters. This method enables a 5-fold higher emitter density while discriminating between fluorophores whose peak emissions are less than 15 nm apart. Our approach can find widespread use in single-molecule applications that rely on distinguishing spectrally different fluorophores under low photon conditions.

Super-resolution microscopy, or nanoscopy, has revolutionized the life and material sciences as it allows surpassing the optical diffraction limit by more than an order of magnitude.^1−4^ One frequently used implementation is single-molecule localization microscopy (SMLM), in which the stochastic activation of single fluorescent emitters leads to spatially separated point spread functions (PSFs) that are used to determine the position of each emitter with sub-50 nm accuracy. Localizations obtained via (direct) stochastic optical reconstruction microscopy ((d)STORM),^1,4^ point accumulation for imaging in nanoscale topography (PAINT),^5−8^ or photoactivated localization microscopy (PALM)^3,9,10^ provide access to detailed structural images or can quantify dynamics and mobilities via single-particle tracking (spt).^10,11^ In this capacity, SMLM has led to breakthroughs^12,13^ in fields such as DNA–protein interactions,^14−17^ cell biology,^18−20^ and soft matter.^8,21−23^

Improving throughput via multiplexing of different fluorophores in SMLM,^5^ enabling microenvironmental characterization,^24,25^ or studying fluorophore-to-fluorophore distance (via single-molecule Förster resonance energy transfer (smFRET)),^26,27^ can be accomplished by combining SMLM with the additional spectral characterization of emitters. Spectral information on single emitters can be acquired and analyzed using various implementations that all rely on placing additional components into the optical detection pathway.

The first implementation of spectrally resolved SMLM uses the ratiometric distinction of spectral emission profiles and is based on placing one (or more) suitable dichroic mirror(s) in the emission pathway.^28−31^ Photons emitted from the sample are separated based on their wavelength and directed toward two different detection channels. This entails either two separate detectors or using two areas on the same camera chip after using additional lenses or mirrors to guide the beams. Then the PSFs and their integrated intensities obtained in the two channels are matched, and the intensity ratio of photons is used to discriminate between the emission spectra of different fluorophores. Importantly, this method requires photons to be directed toward each channel, implicating that only a defined spectral range around the cutoff wavelength of the dichroic mirror can be accessed.

The second implementation uses point spread function engineering to obtain spectral information on emitters. Here, a spatial light modulator (SLM) or a phase mask (PM) is employed in the Fourier plane of the emission path.^32,33^ The introduced phase offset by these elements depends on the incoming wavelength, which can be exploited to design a pattern so that different PSF shapes are realized when photons of different wavelength arrive at the detector. However, small spectral emission differences in the order of tens of nanometers in the peak emission cannot create sufficiently distinct PSF shapes, hindering discrimination of spectrally close fluorophores. Moreover, the voltage (for SLM) or phase (for PM) has to be specifically tuned for certain emission wavelengths, complicating this method when different fluorophores are used.

In the third implementation, spectral dispersion, a spectrally dispersive optical element is added, after which the spatial and spectral profiles are guided to different regions on a single camera chip or to completely separate detectors.^34−41^ Generally, the spatial profile is then analyzed with regular single-molecule localization algorithms,^42−46^ while the spectral profile is spread out over tens of pixels and is used to determine the corresponding emission profile. While this implementation allows a large spectral range to be used and allows the discrimination of fluorophores with similar emission spectra, it has various downsides. First, the entire emission pathway needs to be modified to separate the spatial from the spectral channel.^37,47^ Second, as the spectral information is widened over tens of pixels, the signal-to-noise ratio obtainable in this channel is compromised, leading to a loss in spectral accuracy.^48^ The wide spreading of emission further directly limits the usable density of emitters in the sample, as overlapping spectral profiles cannot be resolved.

In comparing these implementations, it is obvious that there is a need to combine an easy implementation with a broad spectral range and good specificity. Here, we demonstrate spectrally resolved single-molecule localization microscopy (sSMLM) using an inexpensive (blazed) transmission grating that can be easily implemented in most microscope configurations allowing for widespread adoption while maximizing the achievable signal-to-noise ratio. By using a grating with a large line pitch and placing it close to the image plane to minimize the dispersion of the 1^st^ order, we created a low-dispersion sSMLM implementation in which the 0^th^ and 1^st^ diffraction order of every emitter is imaged and analyzed by existing single-molecule localization algorithms in a single field of view without the need for any additional optical elements. This concept can be straightforwardly combined with SMLM methods employing structured excitation profiles^49−54^ and with 3-dimensional PSF engineering approaches,^43,44,55−59^ further increasing the potential use of our implementation. Our novel implementation is capable of accurately determining spectral properties of single molecules at much higher emitter densities than other spectral-dispersing sSMLM implementations. With our sSMLM approach, we show the technical feasibility of spectral multiplexing and ability to distinguish between 0 and 15% FRET efficiency in single-molecule FRET experiments.

To maximize the signal-to-noise ratio and the achievable molecular density in spectrally resolved single-molecule localization microscopy (sSMLM), the available photon budget should be distributed over as few camera pixels as possible.^48^ To fulfill this criterion, we placed a transmission grating with low dispersion (70 lines/mm) as close as possible to a camera chip representing the image plane (<1 cm, Figure 1a) by placing it inside the camera housing. This arrangement minimizes the separation of the 0^th^ and 1^st^ order diffraction patterns and thus results in the highest achievable fluorophore density and signal-to-noise ratio for the 1^st^ order diffraction pattern (Figure 1b,c). Notably, and distinctly different from earlier implementations,^34−37^ our arrangement allows imaging of both spatial and spectral information in the same field of view, thereby maximizing the usable detection area on the sensor. Furthermore, our approach does not require any additional optical components such as mirrors, beam splitters, or secondary detectors.

**Figure 1:**
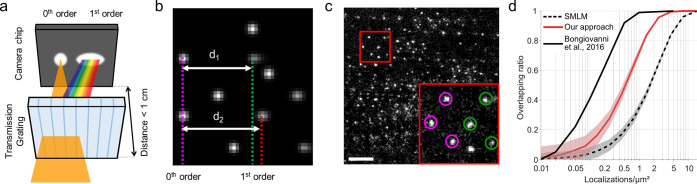
Implementation of low-dispersion spectrally resolved single-molecule localization microscopy. (a) A low-dispersion blazed transmission grating is placed in the emission path of a typical SMLM capable microscope such that the distance of the grating to the image plane is minimized. Around 50% of the light passing the grating will not have any dispersion, causing a 0^th^ order point spread function (PSF) to appear. The other 50% of the light is dispersed based on its wavelength and will create a second, slightly elongated 1^st^ order diffraction pattern. Image not to scale. (b) Simulation of low-dispersion sSMLM data of two spectrally different emitters with λ_1_ (corresponding to *d*_1_) < λ_2_ (corresponding to *d*_2_). Six emitters create in total five 0^th^ and 1^st^ order diffraction pairs on the detector, which can be linked together (two 1^st^ order diffraction patterns are not captured in the field of view). The obtained distances between the 0^th^ and 1^st^ order diffraction patterns (*d*_1_ and *d*_2_) are a measure for the average emission wavelength. (c) Single frame of raw data of a DNA-PAINT nanoruler sample showing 85 spatio-spectrally resolvable emitters in a 31 × 31 μm^2^ field of view. The red outline is enlarged in the inset, in which the 0^th^ and 1^st^ order diffraction patterns are encircled in magenta and green, respectively. Scale bar represents 5 μm. (d) Comparison of achievable emitter density in standard (non-spectrally resolved) SMLM (black dotted line), our approach (red line), and sSMLM with 20–30 pixels spectral pattern elongation, taken from Bongiovanni et al.^37^ The shaded background indicates the standard deviation as determined from repeating the simulations.

As the PSFs from the obtained 1^st^ order diffraction pattern show only minor excess width compared to the PSFs in the 0^th^ order, we were able to employ existing super-resolution algorithms to independently obtain subpixel localizations of the 0^th^ and 1^st^ order diffraction patterns. Next, we linked the localizations with each other in the dispersion direction, with the distance between the 0^th^ and 1^st^ order diffraction patterns (*d*_1_ and *d*_2_ in Figure 1b; further called “0^th^-to-1^st^-order distance”) being a direct measure for the average emission wavelength of the emitter (λ_1_ < λ_2_ with *d*_1_ < *d*_2_). Moreover, the excess width of the 1^st^ order diffraction pattern compared to the width of the PSF in the 0^th^ order is a measure for the width of the emission spectrum. This directly results in a spectral accuracy being limited primarily by spatial single-molecule localization accuracy, an area of research that is progressing very rapidly via both software and hardware developments.^42,55,60^ Using minimal dispersion, the achievable density in our implementation without the need for specialized high-density fitting algorithms is around 5 times higher than earlier implementations of sSMLM, where the spectral information is spread out over 20–30 pixels (Figure 1d; Methods, Supporting Information).^37^

We determined the distance between the dispersion-inducing optics of the grating to the camera chip to be 6.9 ± 0.1 mm in our system (Methods, Supporting Information, Supplementary Figure 1a,b). The spectral dispersion (SD) was determined by calculating the 0^th^-to-1^st^-order distance of a sample labeled with ATTO542 and ATTO655 (Supplementary Figure 1c). From the median distance of these obtained distances and the mean emission profile of the fluorophores, a spectral dispersion of ∼0.21 nm/nm (spatial/spectral; equivalent here to ∼27 nm/px (spectral/spatial)) was determined. We did not observe a wavelength dependency on the angle between the spatial and spectral profiles (Supplementary Figure 1d).

The signal-to-noise ratio of the obtained 1^st^ order diffraction pattern, combined with high resolution of subpixel localization algorithms, indicates that small spectral differences can be elucidated. We imaged double-labeled fixated Cos7 cells, in which clathrin was labeled with the fluorophore CF660, and tubulin with CF680. Pseudocolor coding a super-resolved image based on 0^th^-to-1^st^-order distance reveals good separation of the labeled structures without further analysis (Figure 2a), even though these fluorophores only have a ∼10 nm intensity-weighted spectral separation in our microscope (CF660: 692 nm, CF680: 702 nm, Figure 2b; 12 nm difference in peak wavelength (CF660: 686 nm, CF680: 698 nm)).

**Figure 2:**
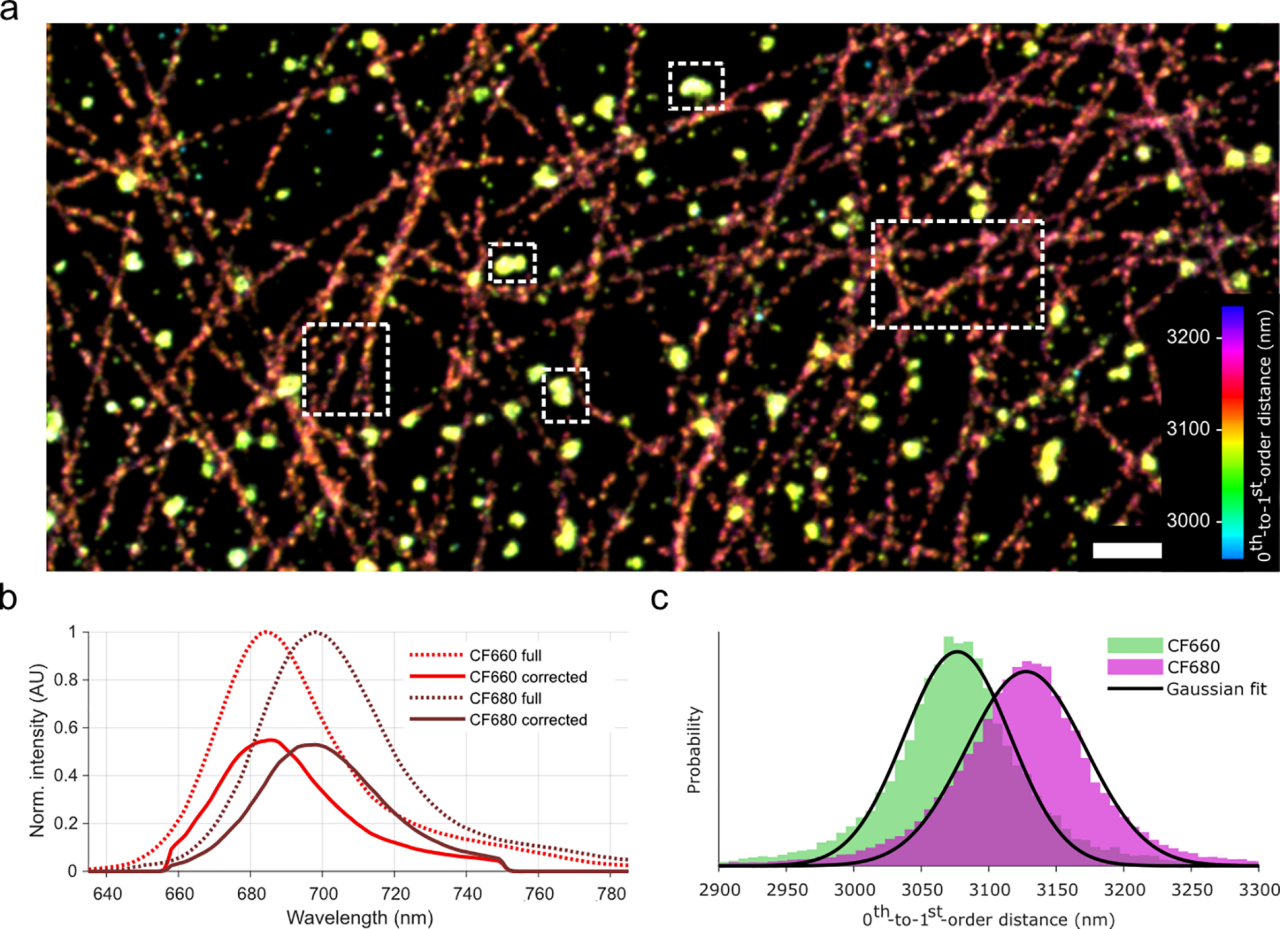
Multiplexing dSTORM of fixated Cos7 cells with CF660-labeled clathrin and CF680-labeled tubules. (a) Obtained dSTORM image, color-coded on 0^th^-to-1^st^-order distance. Separation between tubule and clathrin can be observed without further data analysis. Scale bar represents 1 μm. (b) Emission spectra of CF660 (bright red) and CF680 (dark red). Dotted lines represent full spectra, while the solid lines represent emission spectra corrected for the transmission characteristics of the optical components in the detection path of the microscope. (c) Histograms representing 0^th^-to-1^st^-order distances of fluorophores belonging to areas indicated by dotted outlines in panel a. These populations are fitted with Gaussian curves (see main text) and attributed to CF660 (green) or to CF680 (magenta).

Selecting image regions with mostly CF660- or CF680-labeled structures (dotted outlines in Figure 2a) and fitting the corresponding 0^th^-to-1^st^-order distances with a Gaussian profile reveals that CF660 has a 0^th^-to-1^st^-order distance of 3077 ± 2 nm (σ = 56 ± 2 nm; mean ±95% confidence interval (CI)) and 3128 ± 2 nm for CF680 (σ = 62 ± 2 nm; Figure 2c). This is a difference of 51 ± 2 nm in the raw data, which corresponds to a spectral distance of 10.9 ± 0.4 nm, in agreement with the weighted average and peak position difference of the microscope-corrected emission profiles.

Next, we performed a DNA-PAINT experiment on polystyrene nanoparticles (NPs) that have DNA-PAINT docking strands for either ATTO647N or for ATTO655 (Figure 3a). These fluorophores have a weighted average emission wavelength separated by only ∼9 nm (685 nm and 693 nm, respectively, after correcting for optical components in our microscope, Figure 3b) and a peak emission wavelength separation of ∼14 nm (665 and 679 nm, respectively, after correcting for optical components in our microscope, Figure 3b). After isolating localizations belonging to individual beads and analyzing the 0^th^-to-1^st^-order distances of these emitters, two populations can be observed (Figure 3c).

**Figure 3:**
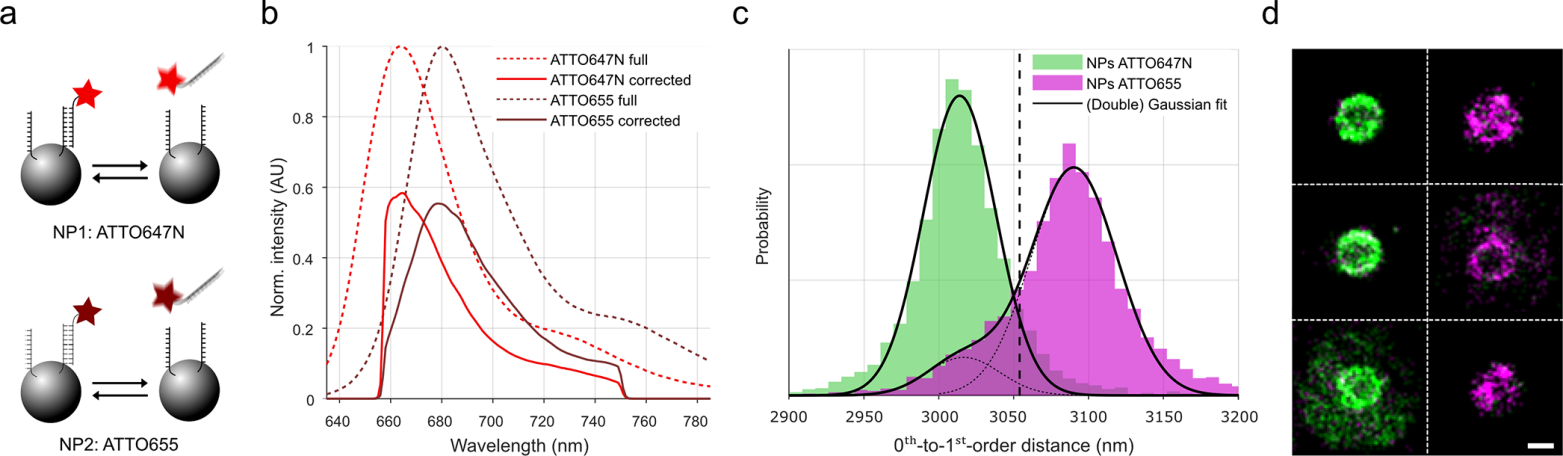
Low-dispersion sSMLM is capable of distinguishing ATTO647N from ATTO655 in DNA-PAINT. (a) Two different nanoparticles have associated DNA-PAINT imager strands containing either ATTO647N or ATTO655. Scheme not to scale. (b) Emission spectra of ATTO647N (bright red) and ATTO655 (dark red). Dotted lines represent full spectra, while the solid lines represent emission spectra corrected for the optical components present in the microscope. (c) Histograms representing observed 0^th^-to-1^st^-order distances of fluorophores belonging to individual NPs. These populations are fitted with Gaussian curve(s) (see main text) and attributed to NPs accepting ATTO647N-DNA (green) or to NPs accepting ATTO655-DNA (magenta). (d) Visualization of six individual NPs, with individual localizations color-coded based on the dotted line shown in panel b. Scale bar represents 500 nm.

The population with the lowest 0^th^-to-1^st^-order distance (green; Gaussian fit peak position: 3014 ± 1 nm, σ = 34 ± 1 nm, mean ±95% CI) was attributed to ATTO647N fluorophores. The population with the larger distances (magenta) was fitted with a combination of two Gaussian curves: one restricted to the fit of the first observed population; along with a unique Gaussian curve (Gaussian fit peak position: 3090 ± 2 nm, σ = 41 ± 3 nm). This population was attributed to ATTO655 fluorophores. The larger standard deviation of the ATTO655 population compared to the ATTO647N population can be attributed to a lower median localization accuracy (42 nm vs 50 nm), possibly caused by a difference in quantum yield (65% vs 30%). The spectral distance between these fitted peak positions (76 ± 2 nm distance; corresponding to 16.2 ± 0.4 nm spectral separation) is close to the difference between the emission peaks of both fluorophores but higher than the weighted average wavelength. This is possibly caused by deviations of the described wavelength-dependent efficiency of optical elements compared to our hardware implementation, which could lead to a shifted weighted mean emission wavelength of ATTO647N, as its emission maximum is close to the lower spectral cutoff (∼660 nm) of the filters and beam splitters used.

Next, all linked localizations were color-coded according to their distance (cutoff at the black dotted line in Figure 3c at 3054 nm). Visualization of the individual NPs (Figure 3d) then reveals their fluorophore distribution. This shows that the NPs are populated by either one DNA-PAINT docking strand or the other, with minimal cross-talk between the used fluorophores, which can be attributed to unspecific DNA–DNA interactions.

Taken together with the dSTORM data of the fixated cells, the obtained order of the mean emission wavelength of these four fluorophores (ATTO647N, CF660, ATTO655, CF680) coincided with that of the 0^th^-to-1^st^-order distance (calculated mean emission wavelength from spectra provided by the manufacturers and corrected for the optical properties of our setup): 685, 692, 693, 701 nm; mean 0^th^-to-1^st^-order distance: 3014, 3077, 3090, 3128 nm). The separation of these 0^th^-to-1^st^-order distances suggests that simultaneous multiplexing of at least three fluorophores with single-wavelength excitation is technically possible under realistic experimental conditions (Supplementary Figure 2).

Next, we were interested whether we could expand the low-dispersion sSMLM to assess single-molecule Förster resonance energy transfer (smFRET). In a typical surface-based smFRET experiment, probes labeled with a donor and an acceptor fluorophore are immobilized and monitored over time. Depending on the experiment, changes in FRET and/or changes in acceptor/donor activity (i.e., blinking and bleaching) can be expected. Conventionally, a ratiometric spectral determination method is applied to separate the donor emission from the acceptor emission on different positions on a camera chip, and the intensity ratio between these is a measure for the relative FRET efficiency E*. In our implementation, we can use the full field of view of our camera and determine E* via the 0^th^-to-1^st^-order distance. We further can utilize the width of the 1^st^ order diffraction pattern as an additional way of discriminating FRET.

We performed smFRET measurements on well-characterized samples of immobilized double-stranded DNA that is dual-labeled with ATTO550 and ATTO647N.^27^ Two samples with different distances between the labeling sites were used: 23-bp separation (∼8.4 nm) and 15-bp separation (∼5.9 nm), leading to apparant FRET efficiencies of ∼0.15 and ∼0.55, respectively. First, we performed simulations using the known emission profiles corrected for the fluorophore’s quantum yield and the optical elements in our microscope (Methods, Supporting Information; Figure 4a,b). These simulations of donor-only, 15% FRET, and 55% FRET samples show that the 0^th^-to-1^st^-order distance follows *d*_donor_ < *d*_15%_ < *d*_55%_, and the width of the 1^st^ order diffraction pattern follows σ_donor_ < σ_15%_ < σ_55%_.

**Figure 4:**
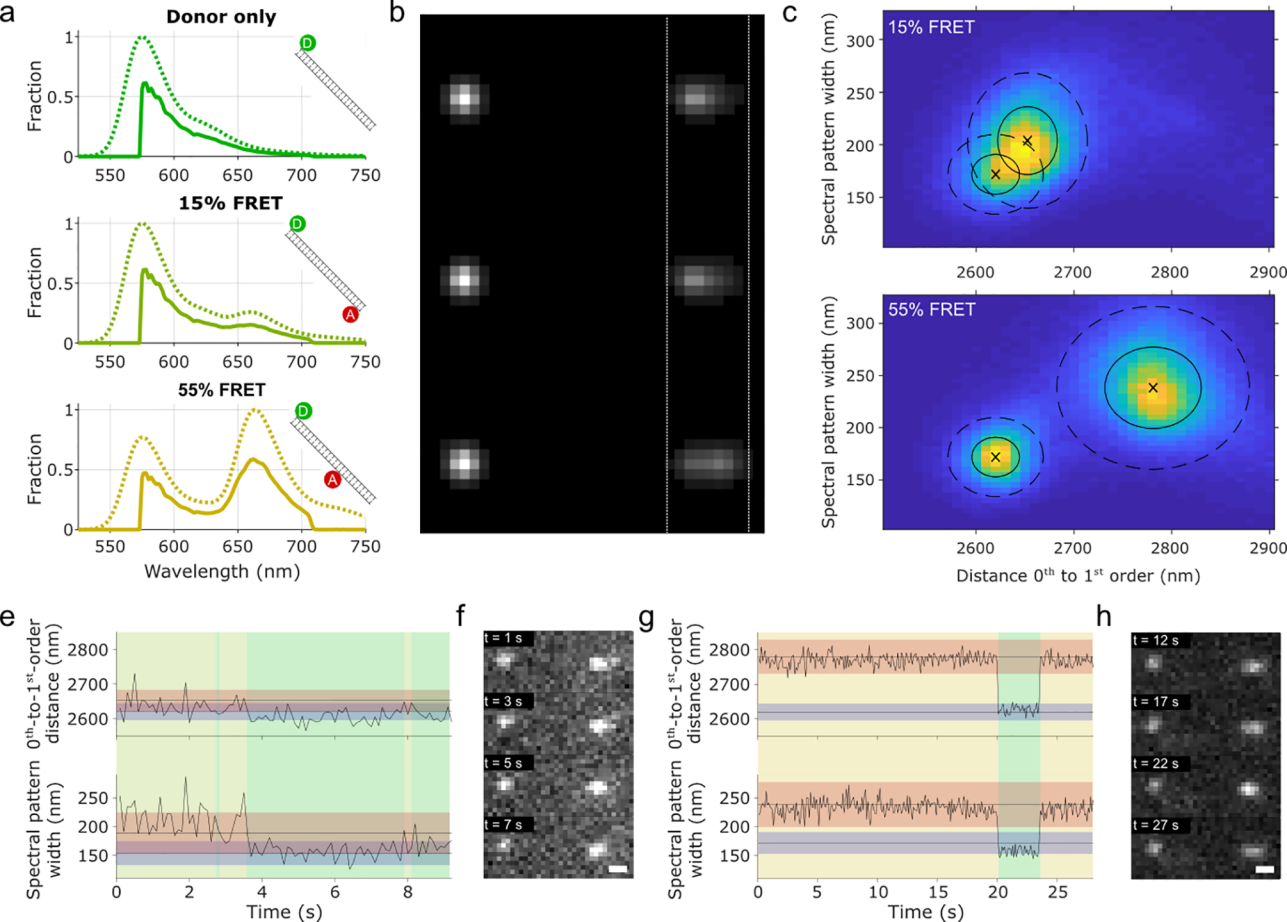
Single-molecule FRET analysis with low-dispersion sSMLM. (a) Predicted emission spectra of donor-only (top, ATTO550), 15% FRET (middle), and 55% FRET (bottom). Dotted lines represent full spectra, while the solid lines represent emission spectra corrected for the transmission characteristics of the optical components present in the microscope. Schemes represent donor (green; ATTO550) and acceptor (red; ATTO647N) fluorophore placements on a DNA strand. (b) Simulated raw data obtained in our low-dispersion sSMLM implementation based on the emission profiles determined in panel a. Vertical dotted white lines are a guide for the eye. (c, d) Two-dimensional histograms of experimental data of (c) 15% FRET and (d) 55% FRET. The histograms were globally fitted with multiple Gaussian functions (shown here centered around black crosses, with solid ellipses representing 1 sigma, and dotted ellipses representing 2 sigma; Methods (Supporting Information), main text). (e–h) Single emitter time trace analysis of (e, f) a bleaching acceptor fluorophore in a 15% FRET pair, and of (g, h) a blinking acceptor fluorophore in a 55% FRET pair. Horizontal gray lines with red and blue shading represent (e) 15% or (g) 55% FRET populations (red) and donor-only populations (blue), determined from the fits in panels c and d. The vertical green, yellow, and orange shading represent current FRET pair state, with green representing donor-only, yellow representing 15% FRET, and orange representing 55% FRET. The raw data corresponding to these FRET pairs throughout the observation time are shown in panels f and h. Scale bars in panels f and h represent 500 nm.

Experimentally, the immobilized DNA strands were imaged separately. Contrary to the multiplexing before, both the 0^th^-to-1^st^-order distance and the width of 1^st^ order diffraction pattern are measures for the FRET efficiency and were therefore visualized (Figure 4c,d). The experimental data agree with the simulations showing *d*_donor_ < *d*_15%_ < *d*_55%_ (2620 nm, 2653 nm, 2781 nm, respectively) and σ_donor_ < σ_15%_ < σ_55%_ (172 nm, 204 nm, 238 nm, respectively).

Next, we explored to which extent we can monitor dynamic behavior using spectrally resolved smFRET. While no direct state transitions are expected for this sample, there is occasional acceptor fluorophore blinking or bleaching, leading to a transition of FRET emission to donor-only emission. For this, we fitted the combined [*d*, σ] 2-dimensional histogram with four 2-dimensional Gaussian profiles (Figure 4c,d black crosses and ellipses). These profiles comprise donor-only, 15% FRET efficiency, 55% FRET efficiency, and “background” states (background state not shown). The “background” state is attributed to nonsense linkages occurring from sparse localizations unrelated to the FRET sample.

Time traces of individual emitters were further assessed (Figure 4e–h). The likelihood of an emitter belonging to the predetermined states was calculated (Methods, Supporting Information), and the most likely state dictated the background color of the graphs in Figure 4e and g. With this methodology, we were able to determine acceptor bleaching (Figure 4e) and acceptor blinking (Figure 4g) in 15% and 55% FRET experimental data. Accurate state determination of the 15% FRET measurement proved to be difficult due to the overlapping Gaussian profiles representing the FRET and donor-only states (Figure 4c), whereas this was better discriminable for 55% FRET.

Here we have demonstrated minimal-dispersion spectrally resolved SMLM (sSMLM), which fundamentally maximizes signal-to-noise and emitter density due to lowest possible photon spread on the detector. In our implementation, we used a single optical component add-on to the detection path leading to a spectral dispersion of just ∼0.2 nm/nm (spatial/spectral), orders of magnitude lower than typical grating-based sSMLM implementations. With this implementation, we realized a five-times increased emitter density compared to similar approaches, achieved good separation of emitters with a ∼10 nm intensity-weighted spectral difference in STORM, and were able to observe changes between 0%, 15%, and 55% FRET efficiency in smFRET.

The low spectral dispersion allows us to use subpixel localization algorithms, a field that is advancing rapidly,^42^ and all future developments are directly applicable to our sSMLM implementation, potentially benefiting from custom deep-learning routines addressing both spectral orders.^56,61^ This could open up avenues for better spatial and spectral precision, more information obtained from the 1^st^ order pattern shape, and sSMLM at even higher emitter density. We additionally note that our implementation can readily be combined with SMLM techniques that modulate excitation patterns to increase localization precision.^49−54^ Finally, low-dispersion sSMLM as presented here can be combined with three-dimensional SMLM by engineering the PSF in the emission path.^57−59^

Work by Song et al. employed subpixel localization algorithms for sSMLM, based on data obtained via the –1^st^ and +1^st^ orders of a nonblazed transmission grating.^38^ While this solution is elegant and uses all photons that arrive on the camera chip for both spatial and spectral localization, blocking out the 0^th^ order leads to a significant loss of photons. Moreover, the implementation requires additional optical components (mirrors and lenses) to direct only the –1^st^ and +1^st^ orders on the camera chip. We additionally note that minimizing the spectral dispersion maximizes spectral precision by minimization of effects by shot-, background-, and read-out noise.^48^

Further minimization of spectral dispersion in our implementation is possible by using a lower dispersion blazed grating by decreasing the effective distance of the grating to the camera chip, if the camera housing permits, or by placing the grating close to an intermediate image plane. While this could result in better spectral resolution and higher achievable sSMLM density, a trade-off of this minimization is decreased information content about the shape of the emission spectrum and the risk of imaging the grating itself on the camera. In this study, we used the shape of the emission spectrum only to discriminate FRET states from the donor-only state.

Taken together, we believe that our implementation of low-dispersion sSMLM will find widespread use due to its inherent simplicity and photon efficiency providing access to maximized spatiotemporal and spectral resolution. We further envision applications in which the photon efficient separation of spectrally different entities is desired such as in low-signal flow cytometry. Here, the ideas taken from super-resolution microscopy such as subpixel localization and spectral peak determination can be equally applied even for low-magnification configurations.

## Notes

The code used in this manuscript can be found on Github, https://github.com/HohlbeinLab/sSMLMAnalyzer. Data underlying the manuscript are available at 10.5281/zenodo.6778964.

## Supporting Information Available

Detailed description of used materials and methods; calibration of low-dispersion sSMLM; technical showcase of triple-fluorophore multiplexing with single-wavelength excitation; ssDNA sequences used for DNA-PAINT on nanoparticles; algorithmic determination of pairing distance and rotation (PDF)

## References

[ref1] Rust M. J.; Bates M.; Zhuang X. Sub-diffraction-limit imaging by stochastic optical reconstruction microscopy (STORM). Nat. Methods 2006, 3, 793 10.1038/nmeth929. 16896339 PMC2700296

[ref2] Hell S. W.; Wichmann J. Breaking the diffraction resolution limit by stimulated emission: stimulated-emission-depletion fluorescence microscopy. Optics Letters 1994, 19, 780–782. 10.1364/OL.19.000780. 19844443

[ref3] Betzig E.; et al. Imaging intracellular fluorescent proteins at nanometer resolution. Science 2006, 313, 1642–1645. 10.1126/science.1127344. 16902090

[ref4] Heilemann M.; et al. Subdiffraction-Resolution Fluorescence Imaging with Conventional Fluorescent Probes. Angew. Chem., Int. Ed. 2008, 47, 6172–6176. 10.1002/anie.200802376. 18646237

[ref5] Jungmann R.; et al. Multiplexed 3D cellular super-resolution imaging with DNA-PAINT and Exchange-PAINT. Nat. Methods 2014, 11, 313 10.1038/nmeth.2835. 24487583 PMC4153392

[ref6] Sharonov A.; Hochstrasser R. M. Wide-field subdiffraction imaging by accumulated binding of diffusing probes. Proc. Natl. Acad. Sci. U.S.A. 2006, 103, 18911–18916. 10.1073/pnas.0609643104. 17142314 PMC1748151

[ref7] Delcanale P.; Miret-Ontiveros B.; Arista-Romero M.; Pujals S.; Albertazzi L. Nanoscale Mapping Functional Sites on Nanoparticles by Points Accumulation for Imaging in Nanoscale Topography (PAINT). ACS Nano 2018, 12, 7629–7637. 10.1021/acsnano.7b09063. 30048592

[ref8] Fuentes E.; et al. PAINT-ing Fluorenylmethoxycarbonyl (Fmoc)-Diphenylalanine Hydrogels. Chem. Eur. J. 2020, 26, 9869–9873. 10.1002/chem.202001560. 32428285 PMC7496660

[ref9] Hess S. T.; Girirajan T. P. K.; Mason M. D. Ultra-High Resolution Imaging by Fluorescence Photoactivation Localization Microscopy. Biophys. J. 2006, 91, 4258–4272. 10.1529/biophysj.106.091116. 16980368 PMC1635685

[ref10] Manley S.; et al. High-density mapping of single-molecule trajectories with photoactivated localization microscopy. Nat. Methods 2008, 5, 155–157. 10.1038/nmeth.1176. 18193054

[ref11] Shen H.; et al. Single Particle Tracking: From Theory to Biophysical Applications. Chem. Rev. 2017, 117, 7331–7376. 10.1021/acs.chemrev.6b00815. 28520419

[ref12] Lelek M.; et al. Single-molecule localization microscopy. Nat. Rev. Methods Primers 2021, 1, 1–27. 10.1038/s43586-021-00038-x. PMC9160414 35663461

[ref13] Liu S.; Hoess P.; Ries J. Super-Resolution Microscopy for Structural Cell Biology. Annu. Rev. Biophys. 2022, 51, 301 10.1146/annurev-biophys-102521-112912. 35119945

[ref14] Uphoff S.; Reyes-Lamothe R.; Garza de Leon F.; Sherratt D. J.; Kapanidis A. N. Single-molecule DNA repair in live bacteria. Proc. Natl. Acad. Sci. U.S.A. 2013, 110, 8063–8068. 10.1073/pnas.1301804110. 23630273 PMC3657774

[ref15] Elf J.; Barkefors I. Single-Molecule Kinetics in Living Cells. Annu. Rev. Biochem. 2019, 88, 635–659. 10.1146/annurev-biochem-013118-110801. 30359080

[ref16] Vink J. N. A.; et al. Direct Visualization of Native CRISPR Target Search in Live Bacteria Reveals Cascade DNA Surveillance Mechanism. Mol. Cell 2020, 77, 39–50.e10. 10.1016/j.molcel.2019.10.021. 31735642

[ref17] Martens K. J. A.; et al. Visualisation of dCas9 target search in vivo using an open-microscopy framework. Nat. Commun. 2019, 10, 3552 10.1038/s41467-019-11514-0. 31391532 PMC6685946

[ref18] Sauer M.; Heilemann M. Single-Molecule Localization Microscopy in Eukaryotes. Chem. Rev. 2017, 117, 7478–7509. 10.1021/acs.chemrev.6b00667. 28287710

[ref19] Leterrier C.; et al. Nanoscale Architecture of the Axon Initial Segment Reveals an Organized and Robust Scaffold. Cell Rep. 2015, 13, 2781–2793. 10.1016/j.celrep.2015.11.051. 26711344

[ref20] Turkowyd B.; Virant D.; Endesfelder U. From single molecules to life: microscopy at the nanoscale. Anal. Bioanal. Chem. 2016, 408, 6885–6911. 10.1007/s00216-016-9781-8. 27613013 PMC5566169

[ref21] Wöll D.; Flors C. Super-resolution Fluorescence Imaging for Materials Science. Small Methods 2017, 1, 1700191 10.1002/smtd.201700191.

[ref22] Martens K.; van Duynhoven J.; Hohlbein J. Spatiotemporal heterogeneity of κ-carrageenan gels investigated via single-particle-tracking fluorescence microscopy. Langmuir 2020, 20, 5502–5509. 10.1021/acs.langmuir.0c00393. PMC7254830 32343144

[ref23] Pujals S.; Feiner-Gracia N.; Delcanale P.; Voets I.; Albertazzi L. Super-resolution microscopy as a powerful tool to study complex synthetic materials. Nat. Rev. Chem. 2019, 3, 68–84. 10.1038/s41570-018-0070-2.

[ref24] Greenspan P.; Fowler S. D. Spectrofluorometric studies of the lipid probe, nile red. J. Lipid Res. 1985, 26, 781–789. 10.1016/S0022-2275(20)34307-8. 4031658

[ref25] Halder A.; et al. Lipid chain saturation and the cholesterol in the phospholipid membrane affect the spectroscopic properties of lipophilic dye nile red. Spectrochimica Acta Part A: Molecular and Biomolecular Spectroscopy 2018, 191, 104–110. 10.1016/j.saa.2017.10.002. 28992460

[ref26] Hohlbein J.; Craggs T. D.; Cordes T. Alternating-laser excitation: single-molecule FRET and beyond. Chem. Soc. Rev. 2014, 43, 1156–1171. 10.1039/C3CS60233H. 24037326

[ref27] Hellenkamp B.; et al. Precision and accuracy of single-molecule FRET measurements—a multi-laboratory benchmark study. Nat. Methods 2018, 15, 669–676. 10.1038/s41592-018-0085-0. 30171252 PMC6121742

[ref28] Purohit A.; Benigno S. P. C.; Richtering W.; Wypysek S. K.; Wöll D. Microgel PAINT–Nanoscopic Polarity Imaging of Adaptive Microgels without Covalent Labelling. Chem. Sci. 2019, 10, 10336–10342. 10.1039/C9SC03373D. 32110321 PMC6984396

[ref29] Baddeley D.; et al. 4D super-resolution microscopy with conventional fluorophores and single wavelength excitation in optically thick cells and tissues. PloS one 2011, 6, e20645 10.1371/journal.pone.0020645. 21655189 PMC3105105

[ref30] Testa I.; et al. Multicolor fluorescence nanoscopy in fixed and living cells by exciting conventional fluorophores with a single wavelength. Biophys. J. 2010, 99, 2686–2694. 10.1016/j.bpj.2010.08.012. 20959110 PMC2956215

[ref31] Gimber N.; Strauss S.; Jungmann R.; Schmoranzer J. Simultaneous Multicolor DNA-PAINT without Sequential Fluid Exchange Using Spectral Demixing. Nano Lett. 2022, 22, 2682–2690. 10.1021/acs.nanolett.1c04520. 35290738 PMC9011399

[ref32] Shechtman Y.; Weiss L. E.; Backer A. S.; Lee M. Y.; Moerner W. E. Multicolour localization microscopy by point-spread-function engineering. Nat. Photonics 2016, 10, 590 10.1038/nphoton.2016.137. 28413434 PMC5391844

[ref33] Broeken J.; Rieger B.; Stallinga S. Simultaneous measurement of position and color of single fluorescent emitters using diffractive optics. Opt. Lett., 2014, 39, 3352–3355. 10.1364/OL.39.003352. 24876051

[ref34] Dong B.; et al. Super-resolution spectroscopic microscopy via photon localization. Nat. Commun. 2016, 7, 1–8. 10.1038/ncomms12290. PMC4962472 27452975

[ref35] Mlodzianoski M. J.; Curthoys N. M.; Gunewardene M. S.; Carter S.; Hess S. T. Super-resolution imaging of molecular emission spectra and single molecule spectral fluctuations. PloS one 2016, 11, e0147506 10.1371/journal.pone.0147506. 27002724 PMC4803349

[ref36] Zhang Z.; Kenny S. J.; Hauser M.; Li W.; Xu K. Ultrahigh-throughput single-molecule spectroscopy and spectrally resolved super-resolution microscopy. Nat. Methods 2015, 12, 935 10.1038/nmeth.3528. 26280329

[ref37] Bongiovanni M. N.; et al. Multi-dimensional super-resolution imaging enables surface hydrophobicity mapping. Nat. Commun. 2016, 7, 13544 10.1038/ncomms13544. 27929085 PMC5155161

[ref38] Song K.-H.; Zhang Y.; Brenner B.; Sun C.; Zhang H. F. Symmetrically dispersed spectroscopic single-molecule localization microscopy. Light Sci. Appl. 2020, 9, 92 10.1038/s41377-020-0333-9. 32509299 PMC7248114

[ref39] Jeffet J.; et al. Multimodal single-molecule microscopy with continuously controlled spectral resolution (CoCoS). Biophys. J. 2022, 121, 301a 10.1016/j.bpj.2021.11.1242. PMC9680784 36425313

[ref40] Kumar Gaire S.; et al. Accelerating multicolor spectroscopic single-molecule localization microscopy using deep learning. Biomed. Opt. Express 2020, 11, 2705–2721. 10.1364/BOE.391806. 32499954 PMC7249819

[ref41] Song K.-H.; et al. Monolithic dual-wedge prism-based spectroscopic single-molecule localization microscopy. Nanophotonics 2022, 11, 1527–1535. 10.1515/nanoph-2021-0541. 35873202 PMC9307059

[ref42] Sage D.; et al. Super-resolution fight club: assessment of 2D and 3D single-molecule localization microscopy software. Nat. Methods 2019, 16, 387 10.1038/s41592-019-0364-4. 30962624 PMC6684258

[ref43] Li Y.; et al. Real-time 3D single-molecule localization using experimental point spread functions. Nat. Methods 2018, 15, 367–369. 10.1038/nmeth.4661. 29630062 PMC6009849

[ref44] Martens K. J. A.; Bader A. N.; Baas S.; Rieger B.; Hohlbein J. Phasor based single-molecule localization microscopy in 3D (pSMLM-3D): An algorithm for MHz localization rates using standard CPUs. J. Chem. Phys. 2018, 148, 123311 10.1063/1.5005899. 29604874

[ref45] Smith C. S.; Joseph N.; Rieger B.; Lidke K. A. Fast, single-molecule localization that achieves theoretically minimum uncertainty. Nat. Methods 2010, 7, 373–375. 10.1038/nmeth.1449. 20364146 PMC2862147

[ref46] Martens K. J. A.; Jabermoradi A.; Yang S.; Hohlbein J. Integrating engineered point spread functions into the phasor-based single-molecule localization microscopy framework. Methods 2021, 193, 107–115. 10.1016/j.ymeth.2020.07.010. 32745620

[ref47] Butler C.; et al. Multi-Dimensional Spectral Single Molecule Localization Microscopy. Front. Bioinform. 2022, 2, 813494 10.3389/fbinf.2022.813494. 36304321 PMC9580959

[ref48] Song K.-H.; Dong B.; Sun C.; Zhang H. F. Theoretical analysis of spectral precision in spectroscopic single-molecule localization microscopy. Rev. Sci. Instrum. 2018, 89, 123703 10.1063/1.5054144. 30599574 PMC6289825

[ref49] Balzarotti F.; et al. Nanometer resolution imaging and tracking of fluorescent molecules with minimal photon fluxes. Science 2017, 355, 606–612. 10.1126/science.aak9913. 28008086

[ref50] Reymond L.; et al. SIMPLE: Structured illumination based point localization estimator with enhanced precision. Opt. Express, 2019, 27, 24578–24590. 10.1364/OE.27.024578. 31510345

[ref51] Gu L.; et al. Molecular resolution imaging by repetitive optical selective exposure. Nat. Methods 2019, 16, 1114–1118. 10.1038/s41592-019-0544-2. 31501551

[ref52] Cnossen J.; et al. Localization microscopy at doubled precision with patterned illumination. Nat. Methods 2020, 17, 59–63. 10.1038/s41592-019-0657-7. 31819263 PMC6989044

[ref53] Jouchet P.; et al. Nanometric axial localization of single fluorescent molecules with modulated excitation. Nat. Photonics 2021, 15, 297–304. 10.1038/s41566-020-00749-9.

[ref54] Gwosch K. C.; et al. MINFLUX nanoscopy delivers 3D multicolor nanometer resolution in cells. Nat. Methods 2020, 17, 217–224. 10.1038/s41592-019-0688-0. 31932776

[ref55] Xu F.; et al. Three-dimensional nanoscopy of whole cells and tissues with in situ point spread function retrieval. Nat. Methods 2020, 17, 531–540. 10.1038/s41592-020-0816-x. 32371980 PMC7289454

[ref56] Nehme E.; et al. DeepSTORM3D: dense 3D localization microscopy and PSF design by deep learning. Nat. Methods 2020, 17, 734–740. 10.1038/s41592-020-0853-5. 32541853 PMC7610486

[ref57] Huang B.; Wang W.; Bates M.; Zhuang X. Three-Dimensional Super-Resolution Imaging by Stochastic Optical Reconstruction Microscopy. Science 2008, 319, 810–813. 10.1126/science.1153529. 18174397 PMC2633023

[ref58] Shechtman Y.; Weiss L. E.; Backer A. S.; Sahl S. J.; Moerner W. E. Precise three-dimensional scan-free multiple-particle tracking over large axial ranges with tetrapod point spread functions. Nano Lett. 2015, 15, 4194–4199. 10.1021/acs.nanolett.5b01396. 25939423 PMC4462996

[ref59] Pavani S. R. P.; et al. Three-dimensional, single-molecule fluorescence imaging beyond the diffraction limit by using a double-helix point spread function. Proc. Natl. Acad. Sci. U.S.A. 2009, 106, 2995–2999. 10.1073/pnas.0900245106. 19211795 PMC2651341

[ref60] Reymond L.; Huser T.; Ruprecht V.; Wieser S. Modulation-enhanced localization microscopy. J. Phys. Photonics 2020, 2, 041001 10.1088/2515-7647/ab9eac.

[ref61] Speiser A.; et al. Deep learning enables fast and dense single-molecule localization with high accuracy. Nat. Methods 2021, 18, 1082–1090. 10.1038/s41592-021-01236-x. 34480155 PMC7611669

