# Supplementary material for: Enabling Spectrally Resolved Single-Molecule Localization Microscopy at High Emitter Densities
Source: Nano Lett. 2022 Oct 21;22(21):8618–25. doi: 10.1021/acs.nanolett.2c03140 (PMC9650776; doi:10.1021/acs.nanolett.2c03140)
Supplement: Supplementary file 1 — nl2c03140_si_001.pdf [file nl2c03140_si_001.pdf]

Supplementary information

belonging to

Enabling spectrally resolved single-molecule localization microscopy at high emitter densities

Koen J.A. Martens[a,b,*], Martijn Gobes[a], Emmanouil Archontakis[c], Roger R. Brillas[c], Niels Zijlstra[a], Lorenzo Albertazzi[c,d], Johannes Hohlbein[a,e,*]

[a] Laboratory of Biophysics, Wageningen University and Research, Stippeneng 4, 6708 WE Wageningen, the Netherlands

[b] *present address*: Institute for Microbiology and Biotechnology, Rheinische-Friedrich-Wilhelms-Universität Bonn, Bonn, Germany

[c] Department of Biomedical Engineering, Institute for Complex Molecular Systems (ICMS), Eindhoven University of Technology, Eindhoven, Netherlands

[d] Nanoscopy for Nanomedicine, Institute for Bioengineering of Catalonia, Barcelona, Spain

[e] Microspectroscopy Research Facility, Wageningen University and Research, Stippeneng 4, 6708 WE Wageningen, The Netherlands

[*] Correspondence: koenjamartens@gmail.com and Johannes.Hohlbein@wur.nl

Materials and Methods

Microscopy hardware for SMLM detection

All measurements were performed on a home-build super-resolution microscope fully described elsewhere[1]. Briefly, the 561 nm and 642 nm laser lines of an Omicron Lighthub 6 (Germany) were employed in HiLo (highly inclined and laminated optical sheet) or TIRF (total internal reflection fluorescence) illumination mode via a Nikon 100x 1.49 NA HP/SR objective (Japan). The emission light was guided via the bypass mode of a rescan confocal microscope (RCM, Confocal.nl, The Netherlands) to an Andor Zyla 4.2+ sCMOS camera (United Kingdom) set to 2 x 2 internal pixel binning leading to an 122 nm effective pixel size. Data acquisition was controlled via micromanager[2]. Either a 405/488/561 dichroic mirror and filter set (ZT405/488/561rpc and ZET405/488/561m-TRF, Chroma, Bellows Falls, VT, USA) for the smFRET experiments, or a 405/488/561/642 dichroic mirror and filter set (ZT405/488/561/642rpc and ZET405/488/561/642m-TRF, Chroma) was used. The second set was only used in experiments where the 642 nm laser line was employed.

Spectral multiplexing with fixated and immunostained cells (dSTORM)

Immobilized Cos7 cells with CF660-immunostained clathrin and CF680-immunostained microtubulin were obtained from Abbelight (France). Cells were imaged for 100.000 frames at 20 ms frame time using 80 mW of laser power at 642 nm excitation wavelength. A STORM buffer (Abbelight, France) was added directly before sealing the sample, which was ~5 minutes before the start of imaging. Data acquisition was started after ~2 minutes of continuous laser illumination used to switch most molecules from an on-state to their off-state thereby lowering the density of emitters.

Spectral multiplexing with polystyrene nanoparticles (DNA-PAINT)

Streptavidin-coated polystyrene nanoparticles (NPs) with a diameter of 400-700 nm (SVP-05-10; Spherotech, Lake Forest, IL, USA) were loaded with single-stranded DNA (ssDNA) by mixing a biotinylated ssDNA strand (Integrated DNA Technologies, Coralville, IA, USA) with the NPs for 1 hr at room temperature, either with docking strand 1 (NP1) or docking strand 2 (NP2)[3]. Two oven-cleaned (heated to 500 °C for 20 minutes to remove organic impurities) precision #1.5H coverslips (Paul Marienfield GmbH, Germany) are used to create a small 'chamber' that is open at two ends, separating the coverslips with double-sided tape. This chamber is filled with a 0.1 mg/mL BSA, 10mM Tris-HCl, 100 mM NaCl, pH 8 solution for 1h, washed with PBS, and filled with PBS containing 0.25 mg/mL NP1, 0.25 mg/mL NP2 for 10 minutes. Afterwards, the chamber is washed with PBS, and 5 nM of each imager strand (complementary to either NP1, containing ATTO655, or complementary to NP2, containing ATTO647N (both Eurofins Genomics, Germany); Supplementary Table 1) in a pH 8 solution containing 5 mM Tris-HCl, 10 mM $MgCl_2$, 1 mM EDTA. The sample is then sealed with tape. Imaging was performed for 2500 frames at 100 ms frame time using a 642 nm laser power of 80 mW in TIRF mode, without any additional emission filter present.

Spectrally-resolved single-molecule Förster resonance energy transfer (smFRET)

Biotinylated double-stranded DNA labelled with ATTO550/ATTO647N with 23-bp (15% FRET) or 15-bp (55% FRET) separation was obtained via a multi-lab study on FRET measurements (1-lo, 1-mid)[4]. PEGylated and biotinylated coverslips were created following an earlier protocol[5]. Briefly, oven-cleaned (heated to 500 °C for 20 minutes to remove organic impurities) coverslips were washed in acetone, and incubated in 1:50 Vectabond/acetone solution (Vector labs, Burlingame, CA, USA). Wells with around 25 µl capacity were created using silicone culture well gaskets (6 mm diameter, Grace Bio-Labs, Bend, OR, USA) , and incubated with 200 mg/ml NHS-PEG (Laysan Bio, Arab, AL, USA) and 2.5 mg/ml NHS-biotin-PEG (Laysan Bio) in 50mM MOPS buffer. Then, ~20 µl 0.02 mg/ml neutravidin was added, and after rinsing with 2x 200 µL PBS, 20 pM of the biotinylated DNA was added. The samples were rinsed with 2x

200 µL PBS, and an oxygen scavenger system was added[6,7] (final concentrations: 1 mM Trolox, 1% glucose oxidase/catalase, 1% glucose). Finally, the gaskets were sealed by placing a coverslip on top. Four movies of 2500 frames each at 100 ms frame time were recorded using ~20 mW laser power (561 nm excitation wavelength) in TIRF mode without an additional emission filter.

Low dispersion diffraction grating for sSMLM

A blazed transmission grating (70 grooves/mm, Edmund Optics, Barrington, NJ, USA, part nr 46-067) was housed in a 3D-printed plastic insert (Supplementary information) and was inserted in the inner tube of the camera. An external C-mount threaded retainer ring (Thorlabs, Newton, NJ, USA, part id CMRR) was then threaded to retain the grating in this place, and the retainer ring was glued to the plastic insert for repeatable insertion of the grating. The grating was inserted such that all diffractions orders from a single emitter are on the same horizontal level. In our implementation, the +1st representing the spectral channel was to the right of the 0th order. Angular deviations from the expected tilt of 0 radians can be accounted for by the software discussed below. As given by the manufacturer, the transmission grating has an overall efficiency of 73% at 632.8 nm wavelength with 41% and 32% for the 0th and +1st order, respectively.

While the absolute distance between the camera chip and the grating is initially unknown, a full rotation of the C-mount thread corresponds to exactly 1/32th inch (~0.8 mm). Four sSMLM experiments with increasingly more full rotations of the grating away from the camera were recorded. The median value of the resulting distance between the 0th and the 1st order is plotted against the absolute distance that the grating has been moved from the closest position (Supplementary Figure 2). Fitting and extrapolating this curve with a 1st order polynomial reveals the distance of the grating to the chip at the crossing of the curve with the *x*-axis.

Analysis of sSMLM data

The dSTORM and DNA-PAINT single-molecule datasets were localized via ThunderSTORM[8] for Fiji[9,10], with the pSMLM plugin[11], after performing a 50-frame temporal median filter[12]. A β-spline wavelet filter with scale 2 and order 3, a local maxima finder with a threshold set to the standard deviation of wave F1 of the filter multiplied by 1.5, and a non-calibrated 3D astigmatism Gaussian fitting routine (dSTORM) or a pSMLM routine with ROI (region of interest) 11-x-11 pixels (DNA-PAINT), respectively, was used. For analyses where the width of the PSFs was critical (i.e. the smFRET experiments), analysis was performed by SMAP with the fit3dSpline fitter[13,14] without a calibrated PSF model. In SMAP, a difference-of-Gaussian filter with size 3 pixels was used alongside an absolute photon cut-off value of 0.3 with a 5-pixel NMS kernel size, to identify PSFs. Fitting was performed with an elliptical PSF with a 13-pixel ROI, 30 iterations. No further filtering was performed.

As the grating leads to PSF pairs representing the 0th order and +1st diffraction order, an algorithm (in MATLAB 2019b (The MathWorks, Natick, MA, USA) or JAVA) needs to identify which pairs belong to an individual emitter. We used a similar analysis as used for linking and analysing individual lobes of a double-helix point spread function[15]. First, possible pairs were found for a given distance and rotation regime from all localizations on a single frame. Rotation and distance bounds can be determined either by hand from the raw images or algorithmically (Supplementary Note 1). Next, localizations that only have a single possible pairing partner were collapsed and refrained from further linking. This is repeated until all pairs are collapsed, or until no further pairs can be collapsed. In the latter case, non-paired localizations were removed from further analysis. Finally, the position of the spatial (here, left-most) localization, the distance and angle between the two localizations, and (if applicable) the obtained PSF width in both dimensions for the spatial and the spectral localizations were stored. The code belonging to this algorithm is provided as supplemental data.

Analysis of spectrally resolved smFRET data

For the spectral smFRET analysis, we used the workflow as presented above while using SMAP with the fit3dSpline fitter for the sub-pixel localisation determination. In addition to the 0th to 1st distances, we obtained the spectral widths of the 1st order of all pairs and plotted this data as a 2D histogram (i.e., spectral distance versus spectral width). The histograms of both 15% and 55% FRET measurements were combined, and fitted with 4 Gaussian profiles, representing donor-only, 15% FRET, 55% FRET, and background populations (not shown) corresponding to spurious or noisy localizations. These Gaussian profiles were re-fitted to either the 15% FRET or the 55% FRET 2D histograms, only changing the relative intensities of the profiles, but not their position or width.

Thereafter, individual linked pairs (i.e., a 0th and 1st order pattern) were classified as 'FRET', 'Donor Only', or 'Background', based on the likelihood of belonging to each Gaussian profile. Individual linked pairs were furthermore linked to other linked pairs throughout time via a simple Nearest-Neighbours tracking algorithm[16], with maximum 1 pixel (122 nm) movement between frames. Only tracks with at least 10 localisations without donor blinking or bleaching events were investigated further.

Simulating sSMLM emission data

Simulation of the expected spatial and spectral diffraction patterns was performed in MATLAB 2019b based on the physical properties of the grating and its placement in respect to the camera. First, the emission spectrum on which the simulation is based is quantised in single wavelength units (i.e., a resolution of 1 nm). For every wavelength, the angle between the 0th and the $+1^{st}$ order diffraction towards the grating is calculated based on the specified density of grooves of the grating (here 70 mm^{-1}). The angle is then used to obtain the spatial position of the 1st order diffraction pattern on the camera chip (based on a specified distance of the grating to the detector). Then, a PSF is approximated via a 2-dimensional Gaussian function, positioned at the 0th order and the 1st order diffraction pattern positions, where the positions are offset by a pre-defined random position between 0 and 1 final pixel size to accurately account for the random positions of emitters in SMLM. The simulated profiles are then normalized with respect to their relative intensity (specified by the emission profile and by the efficiencies of the diffraction orders (41% and 32% for the 0th and 1st order, respectively)). This simulation is first over-sampled on a grid with 1 nm^2-sized pixels, before it is binned into 122 nm by 122 nm pixels, representing condition of our sSMLM hardware. The code belonging to this simulation is provided as supplemental data.

Simulating the resolvable emitter density

The resolvable emitter density possible in our implementation was simulated in MATLAB 2019b with similar conditions as performed previously[17]. A 20-by-20 µm frame was filled with emitters specified by a certain density. Then, localizations that are located closer apart than 3 pixels (here 0.366 µm) are indicated as 'overlapping'. For our sSMLM implementation, a secondary localization was placed to the right of the primary emitter with a randomly chosen distance between 2500 and 3400 nm, and was taken into consideration for overlapping scenarios. This was repeated 500 times at every density to determine the mean and the standard deviation of each condition.

Spectral dispersion characterisation

A 2D DNA-PAINT GATTA-PAINT 80RG (Gattaquant, Germany) sample containing ATTO542 and ATTO655 fluorophores attached to imager strands was imaged with either a 561 nm or a 642 nm laser activated. The 0th-to-1st-order distance was calculated, and the difference in the median distance was divided by the difference of the weighted mean of the fluorophore emission profiles. The fluorophore emission profiles were corrected for the optics and detector (specifically: for the dichroic mirror and filter set, emission filter, and sCMOS quantum efficiency) used in the microscopy system[1].

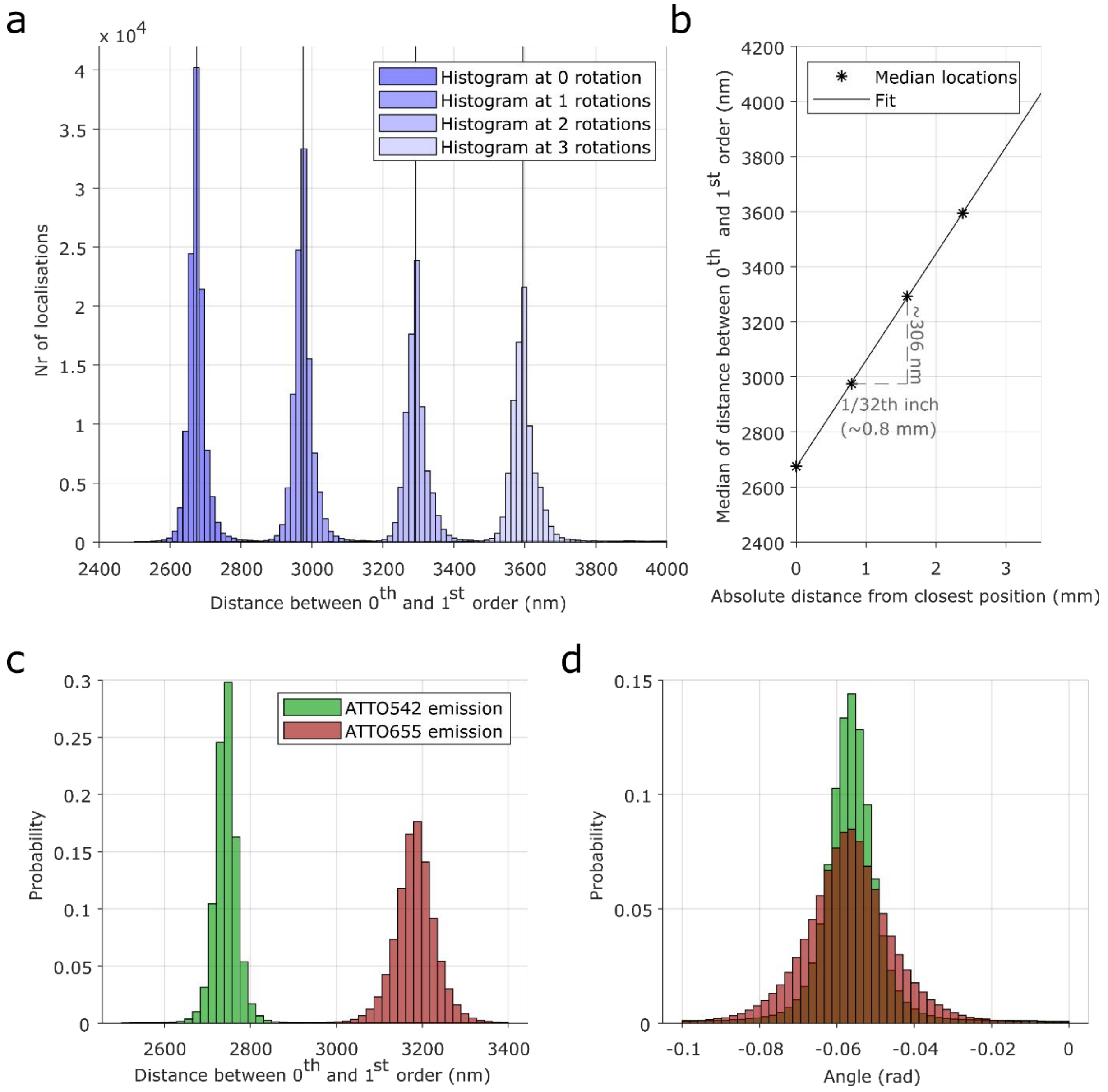

Supplementary Figure 1: *Calibration of low-dispersion sSMLM.* ***a,b)*** *Determination of distance between grating and camera chip via the use of a GATTA-PAINT 80RG DNA-PAINT nanoruler (Methods). The grating is incrementally distanced from the camera by a series of rotations (every rotation is 1/32th inch or ~0.8 mm as specified in the utilized c-mount thread). The histograms of the obtained distances between the 0th and 1st order are plotted in* ***a****, while the linear fit of the median distances is shown in shown in* ***b****.* ***c,d)*** *Determination of the spectral distance (SD). A DNA-PAINT sample with ATTO542 and ATTO655 fluorophores was imaged. The distances (****c****) show a clear difference between the two fluorophores, while the angle (****d****) is not influenced.*

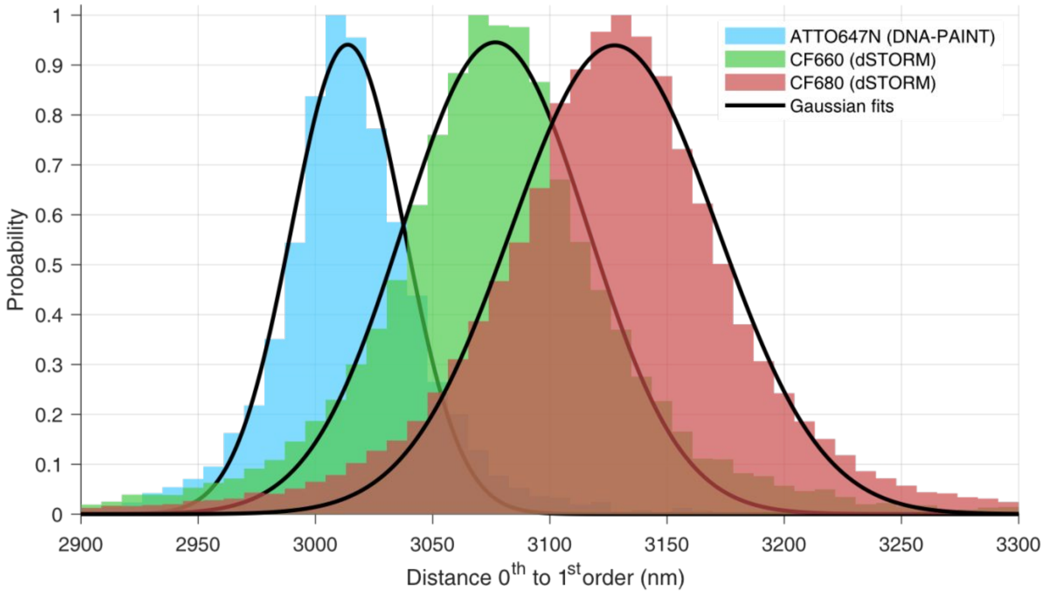

Supplementary Figure 2*: Technical showcase of triple-fluorophore multiplexing with single-wavelength excitation. Combination of the ATTO647N, CF660, CF680 0th-to-1st-order distance data shown in Figures 2 and 3. Rescaled to provide equal probability, and recoloured for clarity.*

Supplementary Table 1: ssDNA sequences used for DNA-PAINT on nanoparticles

Name	Modification	Sequence (5' → 3')
Imager 1	ATTO655 (3' side)	CTA GAT GTA T
Docking 1	Biotin (5' side)	TTA TAC ATC TA
Imager 2	ATTO647N (3' side)	TAT GTA GAT C
Docking 2	Biotin (5' side)	TTA TCT ACA TA

Supplementary Note 1: Algorithmic determination of pairing distance and rotation

The sSMLM pair finding (explained in more detail in the Methods section) requires user-defined limits for the rotation (orientation of the grading with respect to the camera) and for the expected distances between the PSFs. Here, we discuss a JAVA-based implementation to algorithmically determine the average rotation and distance, and determine boundaries required for the pair finding from there. We note that we cannot determine the distance between the grating and the sensor via this approach, and thus all values are effectively the distance between paired PSFs as measured on the sensor.

Briefly, we run a first FFT (Fast Fourier Transformation) on the reconstructed sSMLM images after single molecule localisation (Supplementary Figure. 3a). The first FFT will produce an image as seen in Supplementary Figure 3b. Cropping the image around the center will lead to a figure that shows a near vertical, strip like pattern representing the periodicity of localisations due to the grating (Supplementary Figure 3c). Next, we apply the directionality functionality of ImageJ to obtain the dominant angle in the image representing the orientation of the grating with respect to the sensor. We then apply this angle inversely to rotate the original image, which leads to all pairs of localisations in the 0th and 1st order being horizontally aligned.

Starting from the rotated original image, we perform two consecutive FFTs. The first one will return the stripe-like pattern, now oriented vertically (not shown). The second one will determine the major frequencies representing the two expected distanced for our case of having two different fluorophores in our sample (Supplementary Figure 3d). After cropping (Supplementary Figure 3e) and applying some thresholding (thereby keeping the most intense 0.14% of pixels), we can use ImageJ's particle detection feature to determine the position of the peaks. In Supplementary Figure 3f and g, the main features (#5, #2) to the left of the centre peak (#1) represent the two expected distances between the PSFs of the 0th and the 1st order for the two spectrally distinct fluorophores. The obtained distances will provide the upper and lower estimates for the expected distances then used to run the pair-finding. Here, a secondary angle check is built in as, due to the rotation earlier, the angle of the features to the horizontal should be 0. If this is not the case, a manual check could be necessary. All values are reported to the end user which can then tweak them to process the data using wider or narrower angles/distances.

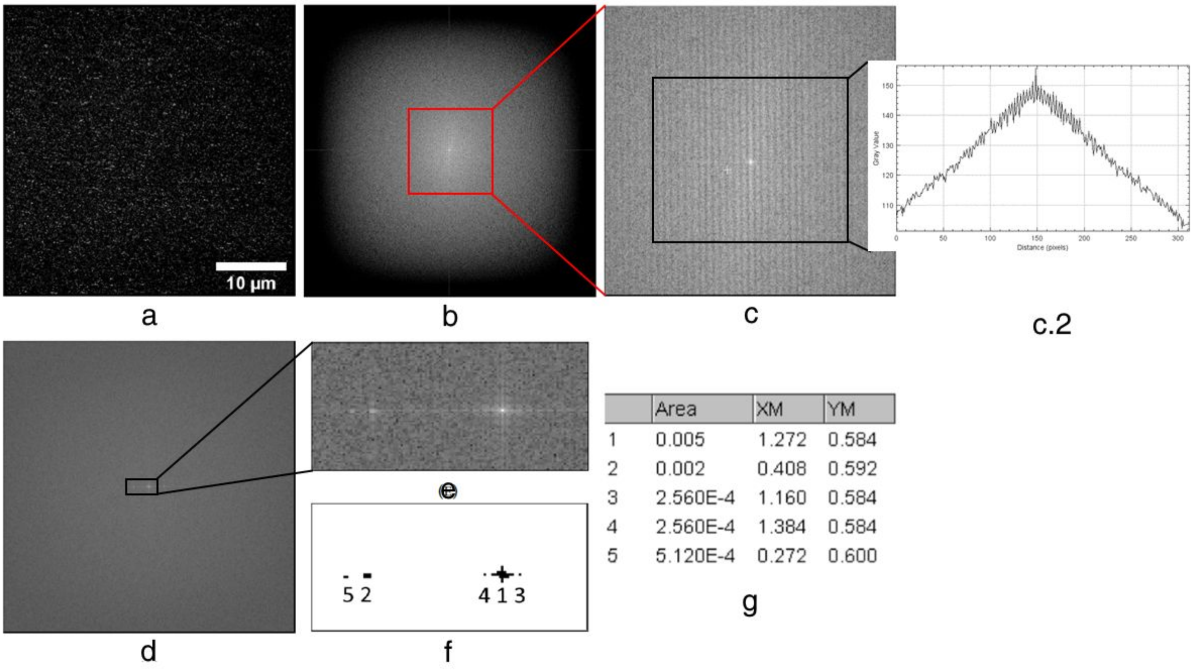

Supplementary Figure 3: *Determining the boundaries for rotation and distances between diffraction orders using Fast Fourier Transformation (FFT).* ***a*** *Super-resolved image of DNA nanorulers (Gattaquant, Germany) with two different fluorophores on the imager DNA strands after analyzing raw data featuring both 0th and 1st order PSFs with ThunderSTORM-phasor.* ***b*** *2D FFT of* ***a*** *indicates periodicity due to the pairing of single emitters in the 0th and +1st diffraction order.* ***c*** *Zoom-in of* ***b****. From this cropped image the angle is determined using the Directionality functionality in ImageJ. An insert shows the summed plot profile over the marked area, indicating periodicity.* ***b*** *2D FFT of* ***c****. The outer edges of* ***b*** *are discarded as they provide no information in the higher frequency k-space and we want to increase the relative magnitude of the pattern.* ***e*** *Zoom-in of* ***d****, showing the only features of high intensity at the centre. The spots correspond to the wavelengths of the pattern shown in c.2* ***f*** *The same data as in* ***e*** *after thresholding showing only sections of high intensity (top 0.14%).* ***g*** *Running the function 'Analyze Particles' in ImageJ/Fiji on F) determines the position of each particle. The distance from the centre peak (#1) to the features (#2 and# 5) determines the distance boundaries. This also allows for a calculation to check if the angle correction was done properly, since the angle from the features to the centre should be 0 compared to the horizontal.*

The algorithm is furthermore described in pseudocode below.

1. Take the square image containing the sup-pixel localised data (e.g., provided as 2D histogram) and rescale to 1024 pixels by 1024 pixels. Normalise the intensities.
2. A forward FFT is performed using ij.plugin.FFT (size of the resulting image is 1024x1024). After every forward FFT we use ImageJFunctions.wrapNumeric to obtain an image in Cartesian coordinates.
3. Using fiji.analyze.directionality.Directionality_with a binrange of 0 to 180 we obtain the dominant angle, as well as the standard deviation.
4. We rotate the FFT image obtained in 2) by the obtained angle. This is done on the ImageProcessor component. This causes some loss of data since we still maintain a square viewport, but these edges are cropped away later.
5. If the standard deviation of the angle is high (>0.2 rad) we set it to 0.2 rad. Conversely, if it is very low (<0.04 rad) we set it to 0.04 rad. The standard deviation is used to find the angle boundaries (see step 6), so limiting their value ensures that we do not envelop too many points or too few points.

6. We set the upper and lower boundaries for the angle: angle - 2.5 * std to angle + 2.5 * std, respectively. This 2.5 value is set empirically.
7. We crop the rotated FFT image around the center from (256, 256) to (767, 767).
8. On this cropped image another forward FFT is performed. The resolution of the resulting image is 512x512. This image is then cropped from (0, 232) to (256, 281).
9. Using the ImageJ Histogram feature, we determine the value of the top 99.86% of pixels (value determined empirically) and set that as a threshold, In a FFT image, a large amount of pixels are usually of low value (due to low amplitude high frequency components, so this only keeps the few higher intensity features). These isolated features represent the 0th order as well as all 1st orders of emitters present.
10. On this thresholded image the ImageJ ParticleAnalyser is run, calculating the centers of mass of all features present, as well as the size of the features.
    a. The values in the list are rescaled to the original dimensions
    b. The distance from the feature to the centre of the image, as well as the angle compared to the horizontal '0' are calculated.
    c. Features that have a distance of less than 1% of the width of the image or over 40% of the width are discarded. This limit is set empirically to discard features stretching the whole image or artefacts very close to the centre of the image.
    d. If the angles calculated are over 0.1 rad the angle was not calculated before and is corrected by the angle detected here.
11. If features are found the closest and furthest distances are selected.
    a. The low distance cut-off is the closest point's distance * 0.9 + 0.375% of the width of the image
    b. The high distance cut-off is the furthest point's distance * 1.1 + 0.375% of the width of the image.
12. These values are then used to filter point combinations for each frame.

Many of the values seen have been chosen empirically and have worked well for our applications. These values can be adjusted in the source-code or a custom version of this algorithm can be implemented to then be passed to the plugin in ImageJ's macro feature.

Supplementary references

1. Martens, K. J. A. *et al.* Visualisation of dCas9 target search in vivo using an open-microscopy framework. *Nat. Commun.* **10**, 3552 (2019).
2. Edelstein, A. D. *et al.* Advanced methods of microscope control using μManager software. *J. Biol. Methods* **1**, e10 (2014).
3. Schnitzbauer, J., Strauss, M. T., Schlichthaerle, T., Schueder, F. & Jungmann, R. Super-resolution microscopy with DNA-PAINT. *Nat. Protoc.* **12**, 1198 (2017).
4. Hellenkamp, B. *et al.* Precision and accuracy of single-molecule FRET measurements—a multi-laboratory benchmark study. *Nat. Methods* **15**, 669–676 (2018).
5. Evans, G. W., Hohlbein, J., Craggs, T., Aigrain, L. & Kapanidis, A. N. Real-time single-molecule studies of the motions of DNA polymerase fingers illuminate DNA synthesis mechanisms. *Nucleic Acids Res.* **43**, 5998–6008 (2015).
6. Cordes, T., Vogelsang, J. & Tinnefeld, P. On the Mechanism of Trolox as Antiblinking and Antibleaching Reagent. *J. Am. Chem. Soc.* **131**, 5018–5019 (2009).
7. Rasnik, I., McKinney, S. A. & Ha, T. Nonblinking and long-lasting single-molecule fluorescence imaging. *Nat. Methods* **3**, 891–893 (2006).
8. Ovesny, M., Křížek, P., Borkovec, J., Švindrych, Z. & Hagen, G. M. ThunderSTORM: a comprehensive ImageJ plug-in for PALM and STORM data analysis and super-resolution imaging. *Bioinformatics* **30**, 2389–2390 (2014).
9. Abràmoff, M. D., Magalhães, P. J. & Ram, S. J. Image processing with ImageJ. *Biophotonics Int.* **11**, 36–42 (2004).
10. Schindelin, J. *et al.* Fiji: an open-source platform for biological-image analysis. *Nat. Methods* **9**, 676–682 (2012).
11. Martens, K. J. A., Bader, A. N., Baas, S., Rieger, B. & Hohlbein, J. Phasor based single-molecule localization microscopy in 3D (pSMLM-3D): An algorithm for MHz localization rates using standard CPUs. *J. Chem. Phys.* **148**, 123311 (2018).
12. Jabermoradi, A., Yang, S., Gobes, M. I., van Duynhoven, J. P. M. & Hohlbein, J. Enabling single-molecule localization microscopy in turbid food emulsions. *Philos. Trans. R. Soc. Math. Phys. Eng. Sci.* **380**, 20200164 (2022).
13. Li, Y. *et al.* Real-time 3D single-molecule localization using experimental point spread functions. *Nat. Methods* **15**, 367–369 (2018).
14. Ries, J. SMAP: a modular super-resolution microscopy analysis platform for SMLM data. *Nat. Methods* **17**, 870–872 (2020).
15. Martens, K. J. A., Jabermoradi, A., Yang, S. & Hohlbein, J. Integrating engineered point spread functions into the phasor-based single-molecule localization microscopy framework. *Methods* **193**, 107–115 (2021).
16. Isaacoff, B. P., Li, Y., Lee, S. A. & Biteen, J. S. SMALL-LABS: Measuring Single-Molecule Intensity and Position in Obscuring Backgrounds. *Biophys. J.* **116**, 975–982 (2019).
17. Bongiovanni, M. N. *et al.* Multi-dimensional super-resolution imaging enables surface hydrophobicity mapping. *Nat. Commun.* **7**, 13544 (2016).
